# Supplementary material for: Chondroitin Sulfate Proteoglycan 4 Provides New Treatment Approach to Preventing Peritoneal Dissemination in Ovarian Cancer
Source: Int J Mol Sci. 2024 Jan 28;25(3):1626. doi: 10.3390/ijms25031626 (PMC10855983; doi:10.3390/ijms25031626)
Supplement: Supplementary file 1 [file ijms-25-01626-s001.zip › ijms-2703222-supplementary.pdf]

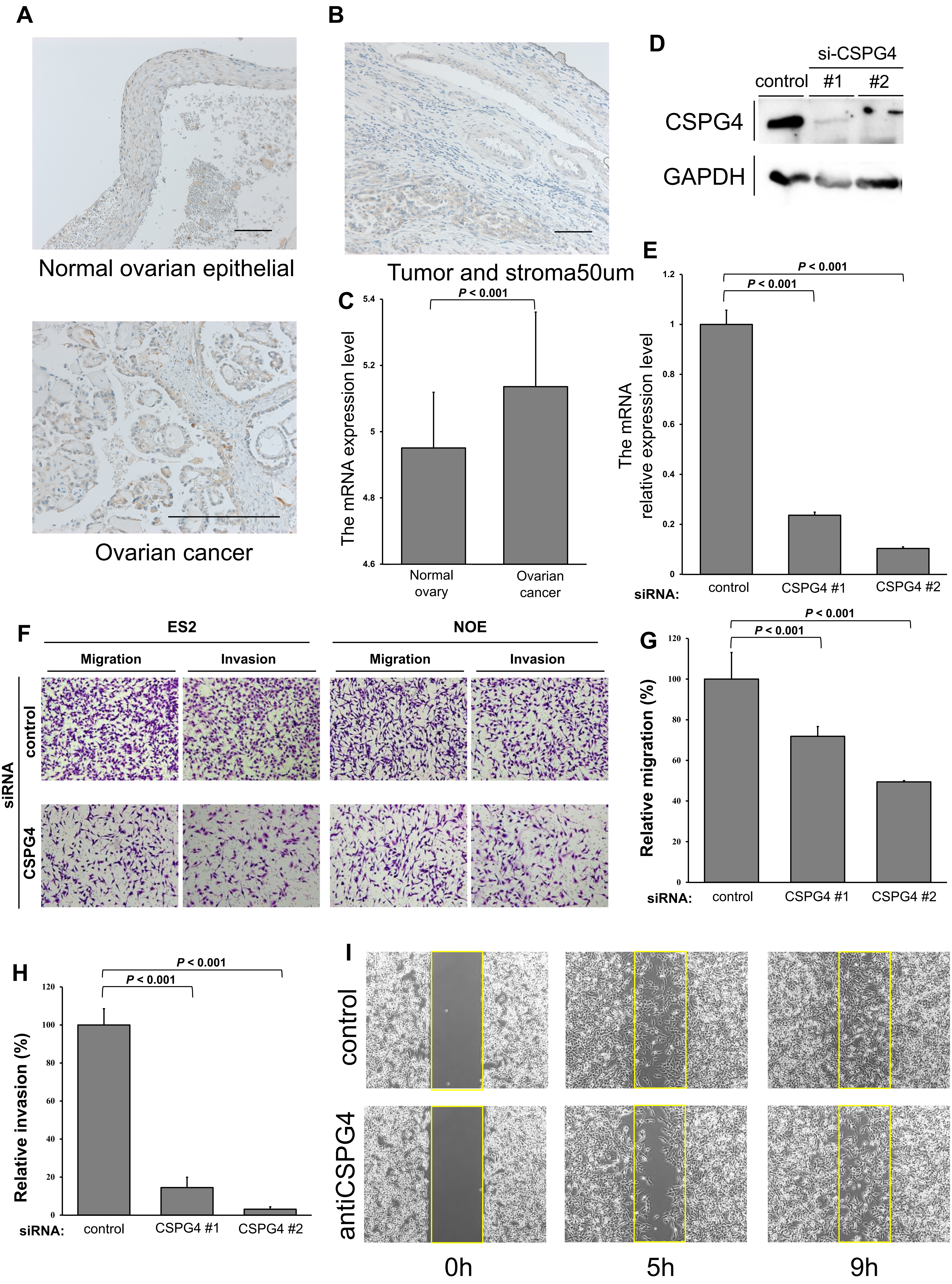

Figure S1. Downregulation of CSPG4 affects invasion, migration, and wound healing abilities in EOC cells

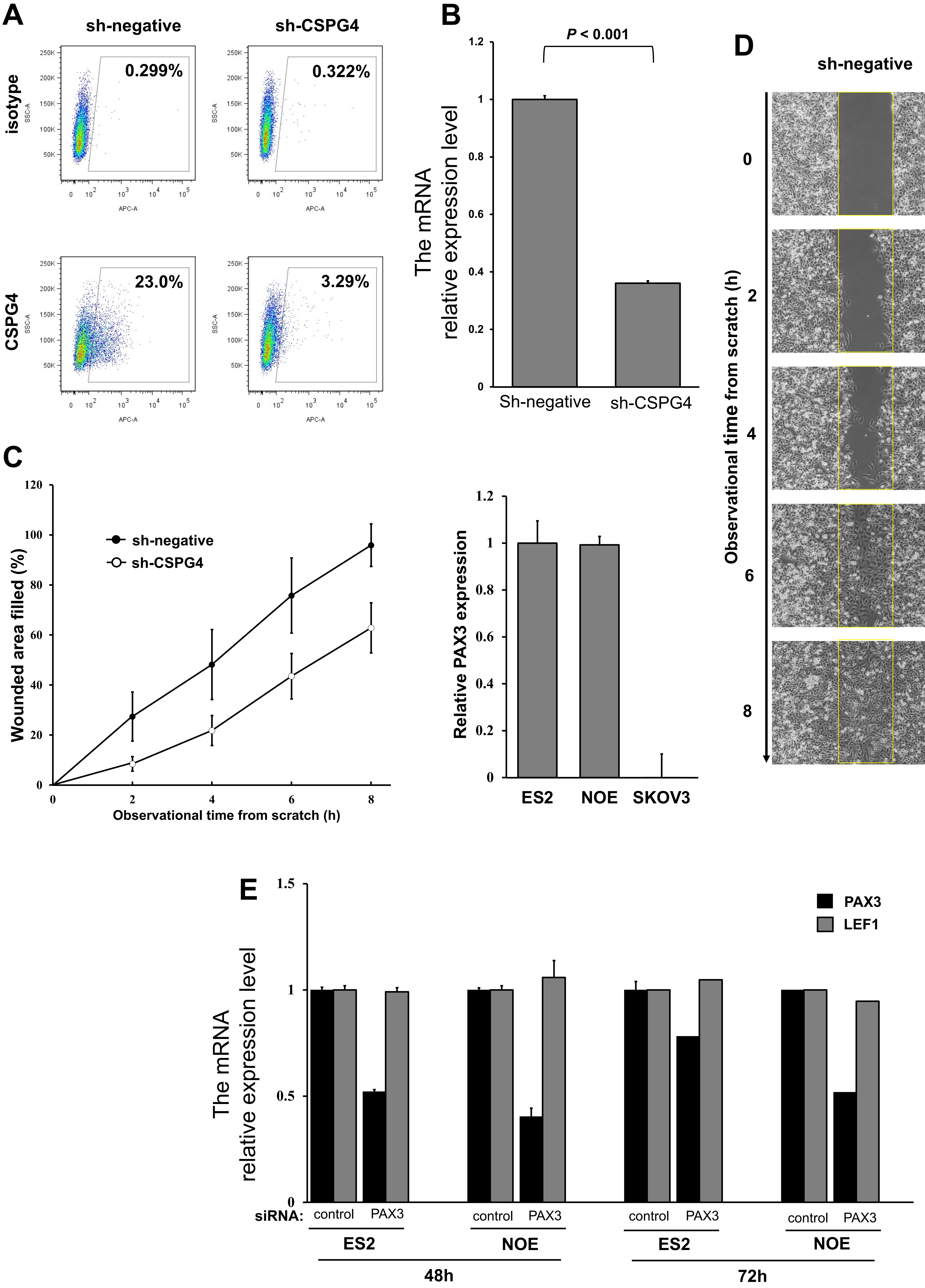

Figure S2. Decreased wound healing ability with sh-CSPG4.

Table S1. Patient backgrounds

Characteristics	All patients (n = 52)
**Age in years, median ± SD (range)**	50 ± 9.2 (26–64)
**FIGO tumor stage**	
II, n (%)	5 (9.6%)
III, n (%)	47 (90.4%)
**Pathology**	
Endometrioid carcinoma, G1, n (%)	1 (1.9%)
Endometrioid carcinoma, G2, n (%)	1 (1.9%)
Endometrioid carcinoma, G3, n (%)	1 (1.9%)
High grade Serous carcinoma, n (%)	39 (75.0%)
Mucinous rcinoma, n (%)	2 (3.8%)
Clear cell carcinoma, n (%)	8 (15.4%)
**Neo-adjuvant chemotherapy, n (%)**	8 (15.4%)

SD, standard deviation; FIGO, International Federation of Gynecology and Obstetrics; G, grade.

Table S2.

Primers

Target gene	primer	Primer sequence (5' to 3')
cspg4	CSPG4-Fw<br>CSPG4-Rev	TGGCCTTCACTGTCACTGTCCTG<br>CAGAGCCTCCGCAGGGATGG
gapdh	GAPDH-Fw<br>GAPDH-Rev	CATGTTCGTCATGGGTGTGAACCA<br>AGTGATGGCATGGACTGTGGTCAT
pax3	PAX3-Fw<br>PAX3-Rev	GCTCCATACGTCCTGGTGCCAT<br>TCTTCTCCACGTCAGGCGTTGTC
lef1	LEF1-Fw<br>LEF1-Rev	CCCCGATGACGGAAAGCATC<br>TGTTCTCGGGATGGGTGGAGAA
fn1	FN1-Fw<br>FN1-Rev	TGTACCATCGCAAACCGCTGCC<br>TGGGCTTGCAGGTCCATTCTCCT

siRNAs

Target gene	siRNA	Target sequence (5' to 3')
non-targeted	control	TTCTCCGAACGTGTCACGT
cspg4	CSPG4 #1	CCAGGTCAACCCTGTCAAT
	CSPG4 #2	GCCGTTCTCTATAGCCACA
lef1	LEF1 #1	GCGATTTAGCTGACATCAA
	LEF1 #2	GCTACATATGCAGCTTTAT

shRNAs

Target gene	shRNA	Target sequence (5' to 3')
non-targeted	control	TTCTCCGAACGTGTCACGT
cspg4	CSPG4-7623	TGGCTTTGACCCTGACTATGTTG
